# An Assessment of Pediatric Residency Applicant Perceptions of "Fit" During the Virtual Interview Era

**DOI:** 10.7759/cureus.31703

**Published:** 2022-11-20

**Authors:** Jason D Vadhan, Jorge G Zarate Rodriguez, Michael Wallendorf, Michael M Awad, Andrew J White

**Affiliations:** 1 Department of Emergency Medicine, University of Texas Southwestern Medical Center, Dallas, USA; 2 Department of Surgery, Washington University School of Medicine, St. Louis, USA; 3 Statistics, Washington University School of Medicine, St. Louis, USA; 4 Department of Pediatrics, Saint Louis University School of Medicine, St. Louis, USA

**Keywords:** residency application, covid-19, residency interviewing, pediatric residency, virtual interviewing

## Abstract

Purpose: Residency recruitment events and interviews are widely considered an integral component of the residency match experience. Due to the COVID-19 pandemic, residency recruitment and interviewing throughout the 2020-2021 academic year were performed virtually, which created challenges for applicants’ ability to discern "fit" to a program. Given this change, it is reasonable to suspect that applicants would be less able to discern program fit. Therefore, this study evaluated how virtual interviews impacted pediatric residency applicants’ ability to assess factors contributing to fit and subsequently how applicants assessed their self-perceived fit to their top-ranked programs.

Methods: An online, anonymous survey was distributed to all residency applicants who applied to any specialty at our large academic institution. The survey utilized a 5-point Likert-type scale to evaluate qualities of fit as well as the applicants’ self-perceived ability to assess these qualities through a virtual platform.

Results: 1,840 surveys were distributed, of which 473 residency applicants responded (25.7% response rate). Among these responses, 81 were pediatric applicants (27.6%). Factors deemed most important in determining fit included how well the residents get along with one another (98.8%), how much the program appeared to care about its trainees (97.5%), and how satisfied residents were with their program (97.5%). Qualities deemed most difficult for applicants to discern included the quality of facilities (18.6%), patient diversity (29.4%), and how well the residents got along with one another (30.2%). When compared to all other residency applicants, pediatric applicants placed more value on whether a program was family-friendly (p = 0.015), the quality of the facilities (p = 0.009), and the on-call system (p = 0.038).

Conclusion: This study highlights factors that influence pediatric applicants’ perception of fit into a program. Unfortunately, many factors deemed most important for pediatric applicants were also among the most difficult to assess virtually. These include resident camaraderie, whether a program cares about its residents, and overall resident satisfaction. Taken together, these findings and the recommendations presented should be considered by all residency program leaders to ensure the successful recruitment of a pediatric residency class.

## Introduction

Residency interviews and recruitment events are important components of the residency match process for program leaders as well as applicants. The COVID-19 pandemic brought about multifaceted challenges in residency recruitment. Among the most notable changes was the implementation of universal virtual interviews for all residency programs nationwide [1-3]. Virtual interviewing has several advantages for residency programs as well as applicants, such as reduced costs for travel and lodging, increased interview scheduling flexibility, and the ability to interview more applicants/programs [3-5]. 

Despite the possibility that virtual interviews may become the norm in the future, there are distinct disadvantages to the process. First, studies have demonstrated the rise of “interview-hoarding” whereby the most competitive applicants interview at the greatest number of programs since there is little incentive to drop interviews [6-9]. This sentiment has been echoed by the Association of American Medical Colleges, which demonstrated that above-average applicants accepted more residency interview offers in the 2020 to 2021 cycle compared to prior years [10]. Prior to virtual interviewing, time and costs hindered interview hoarding. A second notable disadvantage concerns financially disadvantaged applicants, who may lack access to technology or an appropriate virtual interview space, which could result in a worse interview experience, and subsequently a lower rank among residency programs [3,11-13]. Third, with the increase in the number of programs an applicant applies to each year, there is growing concern that programs have less bandwidth to perform a thorough and holistic application review [6,12,14-17]. 

Nonetheless, virtual interviews are becoming the norm within the residency interview process, particularly for large specialties. Unfortunately, virtual interviews come with their own unique set of concerns, notably the notion of “fit” within a program [18]. This is a shared concern for both applicants as well as program directors alike. From the applicant's perspective, assessing a program for fit is consistently rated as one of the most important factors in ranking a program [5,19-21]. This is true across multiple specialties as well as levels of training [5,19,22,23]. Unfortunately, fit is difficult to define, as it is multifaceted and does not currently have a clear or shared meaning [20,21]. Nonetheless, studies have attempted to evaluate applicant fit through a virtual medium, even prior to the COVID-19 pandemic. In 2018, Chandler et al. implemented a pilot virtual interview program for their pediatric surgery fellowship and determined that virtual interviews hindered applicants’ ability to effectively assess program fit and argued against it to replace in-person interviews. Unfortunately, with the development of the COVID-19 pandemic, this became unavoidable. These findings were reproduced among surgery residents as well [19]. 

The primary objective of our study was to explore how the virtual recruitment and interview cycle impacted pediatric residency applicants’ ability to assess fit in programs where they interviewed. Secondarily, we sought to further identify which components of the recruitment and interview process were most important to applicants, and how well applicants could assess these components.

## Materials and methods

An anonymous, online survey was implemented in accordance with Washington University in St. Louis School of Medicine's institutional review board. Participants were identified as those who applied to the following residency programs: dermatology, emergency medicine, general surgery, internal medicine, neurology, neurological surgery, orthopedic surgery, otorhinolaryngology, pediatrics, plastic and reconstructive surgery, physical medicine and rehabilitation, radiology and urologic surgery within a single, large academic institution during the 2020-2021 academic year. Participant email addresses were identified through the institution’s Office of Graduate Medical Education (GME) program-specific residency coordinator. This study is a sub-analysis of previously published data from our institution and compares the responses of applicants to pediatrics to those from other specialties [19]. For this survey, “pediatric specialties” were defined to include only general pediatrics, excluding medicine-pediatrics, pediatric fellowship, or pediatric-psychiatry applicants. The National Residency Match Program (NRMP) was consulted throughout the study preparation phase, and survey implementation was based on their recommendations, specifically with regards to distributing the survey within a timeframe that would reduce applicants’ concerns that the survey might affect their Match, while conversely preventing the Match results from affecting survey responses. As such, surveys were distributed and available for completion immediately following the NRMP Rank Order List (ROL) certification deadline, and ended on Match Day 2021 (a five-day period). Applicants were notified twice more, once halfway through the survey completion period, and once more just before the survey conclusion (one day prior to Match Day 2021). Participants were made aware that all survey questions were optional.

Eighteen unique variables were identified to quantify the notion of fit in accordance with previously published literature, expertise from our institution’s GME, and the NRMP [19,24-26]. First, applicants rated the importance of various factors on their definition of fit, utilizing 5-point Likert-type scales (where 1 = “Not very important for "fit" for me” and 5 = “Very important for “fit” for me”), to what degree survey responders were able to assess these factors throughout virtual interviews (where 1 = “Very difficult to assess” and 5 = “Very easy to assess”), and how effective supplemental virtual recruitment initiatives were in identifying fit (where 1 = “Not helpful at all” and 5 = “Extremely helpful”). Lastly, applicants assessed their personal fit to their top-ranked program using Likert-type scales with paired anchoring statements, whereby higher scores indicated a greater sense of fitting in.

Demographic data such as gender, sexual orientation, race/ethnicity, region of the medical school attended, couples-match status, and preferred specialty was also collected.

Descriptive statistics were performed to summarize the profile of respondents. Pearson’s Chi-Square test was used to identify associations between categorical variables. All data were analyzed using SAS 9.4 (SAS Institute, Cary, NC, USA). We established statistical significance as a p-value < 0.05. “Importance” within pediatric applicant responses was determined as those who responded as either a 4 (important) or a 5 (very important) on the Likert scale within each question.

## Results

A total of 473 residency applicants responded to the survey (overall response rate 25.7%), 81 of which were pediatric residency applicants (pediatrics-specific response rate 27.8%). The survey can be seen in the Supplemental Table in the Appendix.

**Figure 1:**
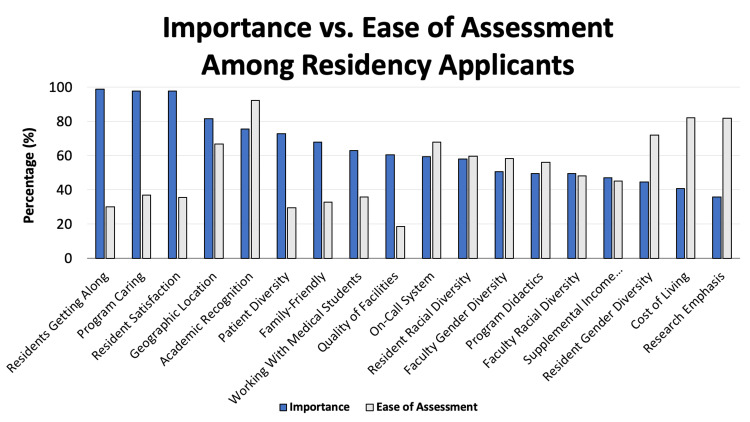
Relative importance versus the ease of assessment among pediatric residency applicants

The most important factors contributing towards perceptions of pediatric residency applicant fit among applicants included how well the residents get along with each other (98.8%), how much the program seems to care about its trainees (97.5%), and how satisfied residents seem with their program (97.5%). Conversely, the least important factors contributing towards pediatric applicants’ perception of fit included research emphasis (35.8%), cost of living (40.7%), and resident gender diversity (44.5%) (Figure 1).

Applicants were also asked to evaluate how easy it was to evaluate these factors via a virtual interview based on their experiences throughout the academic year. The easiest qualities for applicants to evaluate through virtual interviewing included academic recognition of the program (92.0%), cost of living (82.0%), and research emphasis (81.1%). The most difficult qualities to ascertain through virtual interviewing included the quality of the facilities (18.6%), patient diversity (29.4%), and how well the residents get along with each other (30.2%) (Figure 1).

Third, all applicants were instructed to rate the various recruitment activities encountered with respect to how helpful each initiative was in helping the applicant understand the program fit. Applicants rated the interview day (44.7%), the resident-only virtual panel (35.7%), and the residency website's current residents and alumni page (26.8%) as the most helpful factors in discerning fit. Conversely, the programs' social media presence (9.4%), the virtual campus tour (5.7%), and the virtual open house (3.9%) were rated as the least helpful in discerning fit for a program. The most helpful qualities were determined as the highest number of '5' responses on the Likert scale, whereas the least helpful initiatives were determined by the greatest number of '1' responses on the Likert scale (Table 1).

**Table 1:** The Relative Utility of Recruitment Activities in Helping Applicants Discern Program Fit Interventions that were deemed very helpful were given a Likert score of 5, whereas those which were perceived as not helpful were given a Likert score of 1.

Event	Rated Very Helpful (5) %	Rated Not Helpful (1) %
Social Media Presence	8.9%	9.4%
Virtual Campus Tour	11.4%	5.7%
Virtual Open House	13.8%	3.9%
Faculty-Only Virtual Panel	6.0%	3.2%
Resident Curriculum	22.0%	2.5%
Current Residents and Alumni Meeting	26.8%	2.3%
Program Director Question and Answer Meeting	14.9%	1.8%
Program Objectives	25.7%	1.6%
Interview Day	44.7%	0.7%
Resident-Only Panel	35.7%	0.2%

**Figure 2:**
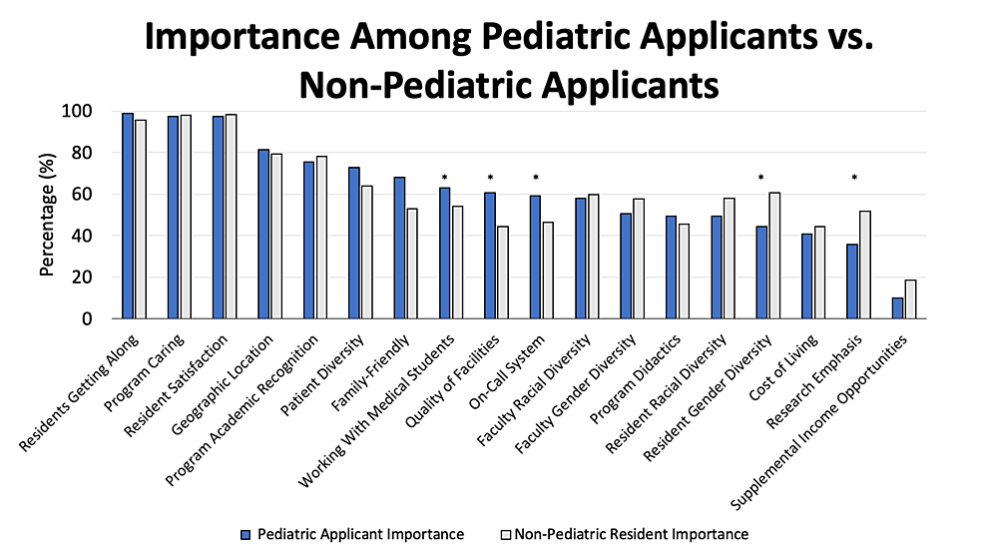
Relative Importance of Residency Program Qualities Among Pediatric Applicants

Last, a comparison between pediatric residency applicants and non-pediatric residency applicants was performed to identify potential differences in qualities that were deemed important to applicants. When compared to the general applicant pool, pediatric applicants placed greater value on whether a program was deemed family-friendly (p = 0.015), the quality of the facilities (p = 0.009), and the on-call system (p = 0.038). Conversely, pediatric applicants placed less value on the resident gender diversity (p = 0.009) and emphasis on research (p = 0.10). Both pediatric and non-pediatric residency applicants placed significant weight on whether residents get along with one another, resident satisfaction, and whether the program appeared to care about their residents. These findings are illustrated in Figure 2. 

## Discussion

The COVID-19 pandemic was a disruptive force across several institutional processes, and the residency interview experience was no exception. The implementation of a virtual interview season is among the most prominent changes and comes in accordance with the Coalition for Physician Accountability [27]. Since the inception of the virtual interview format, there have been a number of studies speculating on its potential consequences [1,3,14,19,28,29]. Our study sought to understand how the virtual residency application process affected medical students’ ability to assess various factors important to applicant fit and their perceptions of their top-ranked pediatric programs.

Qualities that appeared to be most important for pediatric residents included how satisfied current residents seemed with their program, how much the program seemed to care about the residents, and how well the residents get along with one another. These themes appear in alignment with the current shifting landscape of residency towards a more inclusive culture focused on resident wellness [30,31]. Unfortunately, despite the reported importance for applicants to understand how well current residents get along with one another, this was also among the most difficult factors to evaluate through virtual interviewing. These results should come as no surprise since virtual interviewing and recruitment inherently limits the ability of residents to interact with one another while simultaneously hosting an applicant.

Compared to the population at large, applicants applying to pediatrics placed more value on the importance of a family-friendly program and the on-call system. This appears in agreement with a specialty that inherently values a cohesive family unit, as it has been shown to promote healthy childhood development [32,33]. Second, pediatric residency applicants were shown to place less emphasis on resident gender diversity and research. This should similarly come as no surprise, as the pediatric specialty as a whole comprises 64.3% females, which is considerably higher than the national average for gender diversity [34]. Similarly, the emphasis on research also bears little weight among pediatric applicants, which is in concordance across the pediatric specialty, whereby over 70% of graduated residents practice primary care [35]. 

There were several limitations of this study. This survey study capitalized on the unique circumstances posed by the COVID-19 pandemic, which resulted in a rapid shift to a completely virtual interview cycle. Due to the limited timeframe and unprecedented nature of the situation, there was no formal validity testing. However, the survey instrument employed in this study was designed after a thorough literature review and in consultation with a team of research scientists from the NRMP. It was subsequently piloted internally with our institution’s education research group, which is composed of medical education expert faculty, residents of various levels, and medical students. Second, this survey was only distributed to those who applied to a single department, single institution at a large academic center. Given this, the qualities identified as most important and least important may not be generalizable to the population, as there may be specific qualities that led an applicant to apply to this residency program. Future studies should aim at increasing the scope across several institutions across a variety of geographic locations. Third, qualities measured through the survey were selected based on literature review as well as via consultation with several medical education experts, but despite the rigor of question selection, there were additional factors omitted from the survey that was important for applicant fit.

Similarly, and as previously mentioned, the concept of fit is not well-defined in the literature and has been debated for years prior [20,21]. Lastly, it is entirely possible that the same factors reported as being difficult to evaluate through virtual interviewing would have been similarly difficult to evaluate if interviews were in person. Unfortunately, our study does not have a control population to compare this hypothesis. If future residency interviews were to adopt a hybrid model of interviewing, giving the applicant the option to either attend interviews in person or via a virtual platform, future studies could explore and assess differences in the ability to discern perceptions of fit across interview types.

## Conclusions

Our study highlights that many factors deemed most important for pediatric applicants, including resident camaraderie, whether a program cares about its residents, and overall resident satisfaction, are also the most difficult for applicants to assess virtually. This is especially important for residency programs in determining whether future interviews should continue to be held virtually. Although virtual recruitment and interviewing presents several advantages, including increased efficiency, cost-saving, and the ability to interview more candidates, there are also several disadvantages that should be considered, such as the potential for exacerbating inequities between applicants, the reduced ability to perform a holistic review, encouraging application hoarding, and possibly sacrificing a more engaging interview experience. Taken together, these findings should be considered by residency program leaders to ensure successful pediatric residency placement.

## Appendices

**Table 2:** Supplemental Table: How important are the following factors to your definition of “fit”?

	Not very important for "fit" for me 1	2	3	4	Very important for "fit" for me 5
1. Academic recognition of the program					
2. Cost of living					
3. Diversity of the patient population					
4. Emphasis on research					
5. Emphasis on working with medical students					
6. Gender diversity of the faculty					
7. Gender diversity of the residents					
8. Geographic location					
9. How much the program seems to care about its trainees					
10. How family-friendly the program appears to be					
11. How satisfied residents seem with their program					
12. How well the residents get along with each other					
13. On-call system structure and/or frequency					
14. Program didactics/education conference					
15. Quality of the facilities					
16. Racial/ethnic diversity of the faculty					
17. Racial/ethnic diversity of the residents					
18. Supplemental income (moonlighting) opportunities					

	Very difficult to assess 1	2	3	4	Very easy to assess 5
1. Academic recognition of the program					
2. Cost of living					
3. Diversity of the patient population					
4. Emphasis on research					
5. Emphasis on working with medical students					
6. Gender diversity of the faculty					
7. Gender diversity of the residents					
8. Geographic location					
9. How much the program seems to care about its trainees					
10. How family-friendly the program appears to be					
11. How satisfied residents seem with their program					
12. How well the residents get along with each other					
13. On-call system structure and/or frequency					
14. Program didactics/education conference					
15. Quality of the facilities					
16. Racial/ethnic diversity of the faculty					
17. Racial/ethnic diversity of the residents					
18. Supplemental income (moonlighting) opportunities					

	1	2	3	4	5	
I cannot picture myself here						I can picture myself here
I do not feel welcome here						This place feels like home
There are no people like me here						I feel there are people like me here
This program is not a good fit for me						I feel like I fit in here well

	Not at all helpful 1	Helped a little bit 2	Helped somewhat 3	Helped very much 4	Extremely helpful 5	Not offered, or I did not participate
1. Virtual open house						
2. Virtual campus tour						
3. Residents-only virtual panel						
4. Faculty-only virtual panel						
5. Virtual Q&A session with program director						
6. Interview day						
7. Social media presence (Twitter, Instagram, Facebook)						
8. Website – Program structure/objectives						
9. Website – Resident Curriculum						
10. Website – Current Residents and Alumni						
